# Report of the Chicago Retreat for the Insane

**Published:** 1848

**Authors:** 


ARTICLE II.

*Report of the Chicago Retreat for the Insane.* By Edward Mead, M.D., Superintendent. Chicago: R. L. Wilson, Printer. 1848. (From the Author.)

In August last this institution was opened by the reception of one patient, which was speedily followed by others, so that in the six months that elapsed prior to the issue of the report before us, fifteen patients had been admitted; five of these have been cured, one died of organic disease which existed before admission, and nine remained in the institution at the time of the issue of the report.

The practicability of carrying on, advantageously, institutions of this kind by individual enterprise, has by some been doubted, but the determination of Dr. Mead, we hope, will be attended with such success as to convince such of the error of the position they have assumed. Chicago is certainly a most favorable point for an undertaking of the kind, and we shall hope from time to time to hear of the prosperity of the Retreat. For the information of those that may wish to patronize the establishment, we extract the following, giving terms, &c.:

"The terms are $4 per week. For those who desire a separate attendant, and a suit of rooms fitted up in more costly style than the general arrangements, $10 per week. Payment for three months required in advance, and security for monthly payments in advance thereafter. No patient will be admitted for a shorter period than three months. If, however, a patient should recover in a shorter period, such sum as may be agreed upon will be refunded."

